# Serial Interval and Generation Interval for Imported and Local Infectors, Respectively, Estimated Using Reported Contact-Tracing Data of COVID-19 in China

**DOI:** 10.3389/fpubh.2020.577431

**Published:** 2021-01-08

**Authors:** Menghui Li, Kai Liu, Yukun Song, Ming Wang, Jinshan Wu

**Affiliations:** ^1^Beijing Institute of Science and Technology Information, Beijing, China; ^2^Beijing Science and Technology Information Strategy Decision-Making Consultant Center, Beijing, China; ^3^Faculty of Geographical Science, Beijing Normal University, Beijing, China; ^4^School of Systems Science, Beijing Normal University, Beijing, China

**Keywords:** COVID-19, generation interval, imported infection, local infection, serial interval

## Abstract

The emerging virus, COVID-19, has caused a massive outbreak worldwide. Based on the publicly available contact-tracing data, we identified 509 transmission chains from 20 provinces in China and estimated the serial interval (SI) and generation interval (GI) of COVID-19 in China. Inspired by different possible values of the time-varying reproduction number for the imported cases and the local cases in China, we divided all transmission events into three subsets: imported (the zeroth generation) infecting 1st-generation locals, 1st-generation locals infecting 2nd-generation locals, and other transmissions among 2+. The corresponding SI (GI) is respectively denoted as ${\text{SI}}_{1}^{0}$(${\text{GI}}_{1}^{0}$), ${\text{SI}}_{2}^{1}$ (${\text{GI}}_{2}^{1}$), and ${\text{SI}}_{3+}^{2+}$(${\text{GI}}_{3+}^{2+}$). A Bayesian approach with doubly interval-censored likelihood is employed to fit the distribution function of the SI and GI. It was found that the estimated ${\text{SI}}_{1}^{0}=6.52\;(95\%\;\text{CI}:5.96-7.13)$, ${\text{SI}}_{2}^{1}=6.01\;(95\%\text{CI}:5.44-6.64)$, ${\text{SI}}_{3+}^{2+}=4.39\;(95\%\;\text{CI}:3.74-5.15)$, and ${\text{GI}}_{1}^{0}=5.47\;(95\%\;\text{CI}:4.57-6.45)$, ${\text{GI}}_{2}^{1}=5.01\;(95\%\;\text{CI}:3.58-7.06)$, ${\text{GI}}_{3+}^{2+}=4.25\;(95\%\;\text{CI}:2.82-6.23)$. Thus, overall both SI and GI decrease when generation increases.

## 1. Introduction

As of April 21, 2020, COVID-19 has broken out in 213 countries, areas or territories, and the World Health Organization (WHO) has reported over 2, 356, 414 confirmed cases and over 160, 120 confirmed deaths (1). It is a huge challenge to plan intervention strategies aimed at controlling outbreaks of COVID-19 in all countries, areas, or territories (2). Some key disease transmission parameters, including the basic reproduction number, the time-varying reproduction number, the generation interval (GI, time difference between being infected and infecting others), the serial interval (SI, the time difference between symptom onset of the infector and the infectee), and the incubation period (IP, the time difference between being infected and symptom onset), might offer insightful information of the epidemic and thus, might be helpful in devising interventions. In particular, the basic and time-varying reproduction numbers are good indicators of the speed of disease spread and the effectiveness of interventions. The estimation of the basic and time-varying reproduction number often requires SI. In fact, for epidemics that are infectious during the incubation period, estimation of the reproduction numbers requires GI (3). It is possible that COVID-19 is infectious during incubation period (4, 5). Therefore, in this work, we will perform a statistical analysis of GI and SI.

Several papers have quantified the GI, SI, and IP of COVID-19 by employing statistical and mathematical modeling (3, 6–21). Please see Table 1 for their estimated values and sample sizes. It has been found that the estimated values of SI from those previous studies have a wide range: 3.95-7.5 days. However, an accurate estimation of SI (and GI) is crucial in calculating the reproduction numbers accurately. Therefore, in this work, we first want to provide a more accurate estimation of SI (and GI) with possibly larger sample sizes. Second, if possible, we also want to shine some light on why there can be such larger differences in the estimated value of SI.

**Table 1:** Estimated values for serial interval, generation interval, and incubation period in previous papers.

**Interval**	**Mean [95 CI%]**	**SD [95 CI%]**	**Samples (N)**	**References**
SI	3.95 [−4.47–12.5]	4.24 [4.03–4.95]	114^b^^*^	(3)
SI	3.96 [3.53–4.39]	4.75 [4.46–5.07]	486	(10)
SI	4.22 [3.43–5.01]	–	135^b^^*^	(11)
SI	4.4 [2.9–6.7]	3.0 [1.8–5.8]	21	(7)
SI	4.41	3.17	71	(8)
SI	4.56 [2.69–6.42]	–	93^a^^*^	(11)
SI	4.7 [3.7–6.0]	2.9 [1.9–4.9]	28	(9)
SI	4.8	–	112^*^	(18)
SI	5.1 [1.3–11.6]	–	35	(14)
SI	5.21 [−3.35–13.94]	4.32 [4.06–5.58]	91^a^^*^	(3)
SI	5.83	3.58	9	(16)
SI	6.37	4.15	57	(19)
SI	6.6	–	12	(17)
SI	6.70 [6.31–7.10]	5.20 [4.91–5.46]	689	(22)
SI	7.5 [5.5–19]	3.4	6	(6)
GI	5.2 [3.78–6.78]	1.72 [0.91–3.93]	91^a^^*^	(3)
GI	3.95 [3.01–4.91]	1.51 [0.74–2.97]	114^b^^*^	(3)
IP	3.9	–	25	(17)
IP	4.8[2–11]	–	–	(12)
IP	5.0 [4.2–6.0]	3.0 [2.1–4.5]	52	(13)
IP	5.2 [1.8–12.4]	–	49	(14)
IP	5.2 [4.1–7.0]	–	10	(6)
IP	6.4 [5.6–7.7]	2.3 [1.7–3.7]	88	(15)
IP	7.1 [6.13–8.25]	–	93^*a*^	(11)
IP	7.44 [7.10-7.78]	4.39 [3.97–4.49]	587	(22)
IP	8.06 [6.89–9.36]	–	93	(19)
IP	9 [7.92–10.2]	–	135^*b*^	(11)
IP	10.91	–	67	(8)

a *Singapore*.

b *Tianjin, China*.

* *Indicates that there is no number of pairs given in the reference and we then list the number of cases in their datasets instead*.

Another motivation for this work comes from the extended framework of estimating the time-varying reproduction number of COVID-19 in China (Song et al., under review). When working on determining the time-varying reproduction number of COVID-19 in China, we note that due to the different interventions for imported cases and local cases, their time-varying reproduction number should be different. All previous analyses, as far as we know, have assumed that they are the same. See, for example, EpiEstim 2 (23), which is a well-known R software for the estimation of a time-varying reproduction number. For that, we need to distinguish between the reported cases, the zeroth-generation imported cases *X*^0^, the first-generation locals infected by the imported cases *X*^1^, and so on, such as *X*^2^ and *X*^3+^. From the transmission chains among those cases, we then find SI and GI between various generations, such as ${\text{SI}}_{n+1}^{n}$ and ${\text{GI}}_{n+1}^{n}$, the SI and GI between the *n*th generation and the (*n* + 1)th generation.

There are three tasks in the above motivations, namely, obtaining more reliable estimates of GI and SI of COVID-19, finding possible reasons for considerable differences in previous appraisals, and also providing GI and SI for various generations. To accomplish all three of the tasks listed above, we extracted data from online reports released by 20 provincial health commissions in China, except for Hubei province. From that data, we identified 509 transmission chains and estimated transmission parameters. As shown later, the SI and GI for various generations are indeed quite different.

## 2. Materials and Methods

We collected our data from publicly available official reports of case investigations published by provincial/municipal health commissions in China. The case investigations were performed by investigators in the corresponding centers for disease control and prevention in each province. The details of each confirmed case include the following necessary information: case ID, gender, age, date of symptom onset, date of diagnosis, history of traveling to or residing in Hubei or cities other than reporting city, date of arriving at the city where the case is reported. If identified via contact tracing performed by centers for disease control and prevention officers, the details also include contact case ID and date of exposure. The data includes 4, 111 confirmed cases that were compiled from online reports from 20 provinces in mainland China, except for Hubei province, between 21 January 2020 and 29 February 2020. Moreover, the cases are classified into different groups according to travel or residency history and chains of transmission of infection, if data on the case allows, as follows:

1. Imported cases (*X*^0^): Cases known to be infectors outside of Hubei but known to come out from Hubei recently,
2. Local first-generation cases (*X*^1^): Cases known to have been infected by the imported cases,
3. Local second-generation cases(*X*^2^): Cases known to have been infected by the local first-generation cases,
4. Local third-plus cases (*X*^3+^): Cases known to have been infected by local second or higher generation cases.

Imported cases can be Hubei residents who have been living in Hubei for a long time, or Hubei travelers who traveled to Hubei very recently as long as they just came from Hubei recently and became infectors in other provinces in China. The date of symptom onset is defined as the date of appearance of symptoms relevant to COVID-19. The exposure date, which is needed for estimating GI, is estimated to be the middle dates for the earliest and latest possible exposure time for local cases and also for Hubei travelers. For Hubei residents, their exposure dates are hard to find due to the lack of our data on Hubei cases. Therefore, whenever the exposure date was needed, we discarded the data on Hubei residents. We processed the interval-censored data in units of days and discarded non-positive values, which means, in the case of SI, the infector shows symptoms latter than the infectee. This assumption might well be true or be due to some errors in data collection, especially when the infector and the infectee are from the same household. We did find many cases with non-positive values that are from the same household. We decided not to use those non-positive data since it is hard to tell who the infector is between pairs in the same household. Finally, we obtained 509 COVID-19 transmission events, and we named this dataset as “All.” Then, we divided the “All” data into three subsets: Imported-first subsets $E_{1}^{0}$, local first-second subsets $E_{2}^{1}$, local second-third plus subsets $E_{3+}^{2+}$. $E_{1}^{0}$ are composed of the events that imported cases *X*^0^ infect local first-generation cases *X*^1^, and others are defined accordingly.

A report on the data together with the generation labels will be published elsewhere; prior to their publication, a very rough version can be obtained at our project page on GitHub: https://github.com/Bigger-Physics/COVID19-si.

From these transmission chains, we obtained SI and GI for various generations. A Bayesian approach with doubly interval-censored likelihood (24) was then employed to obtain estimates of serial interval distribution, generation interval distribution, and incubation period distribution using the CmdStan (9) package in R.

## 3. Results

### 3.1. Serial Interval

**Figure 1:**
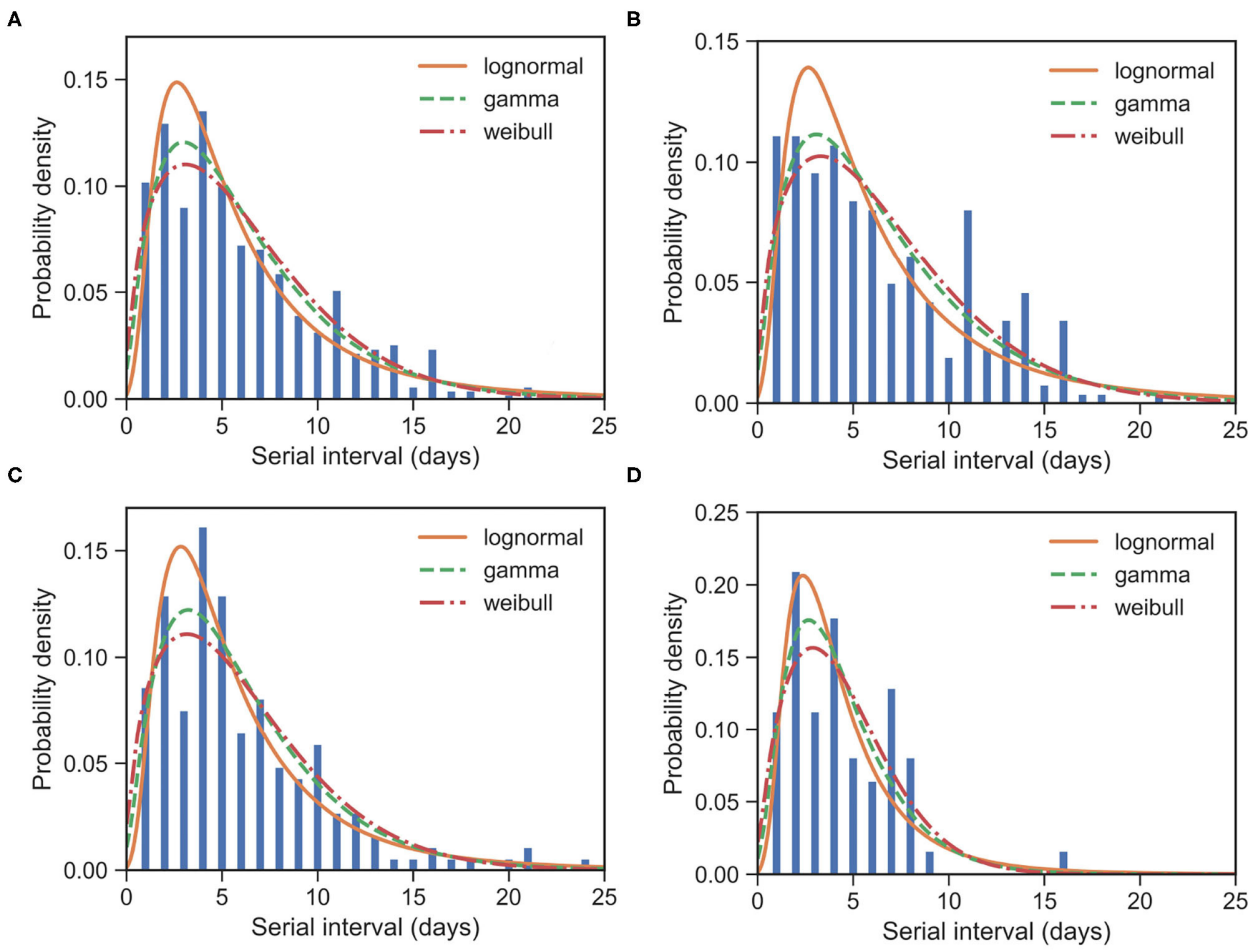
Fitted SI distribution for COVID-19 based on 509 reported transmission pairs in China between 21 January 2020 and 29 February 2020. Bars indicate the empirical distribution of SI samples and lines indicate the fitted lognormal, gamma, and Weibull distributions, respectively. **(A)** The “All” dataset (*N* = 509). **(B)** The imported-first subset $E_{0}^{1}$ (*N* = 261). **(C)** The local first-second subset $E_{1}^{2}$ (*N* = 186). **(D)** The local second-third Plus subset $E_{2+}^{3+}$ (*N* = 62). Values of the fitted parameters can be found in Table 2.

For all 509 samples, the observed SIs have a mean at μ_SI_ = 6.05 days and a standard deviation (SD) at δ_SI_ = 4.28 days. By using all these 509 samples, we estimated the mean at 6.05 (95% CI:5.68 − 6.44) days and SD at 4.32 (95% CI:3.97 − 4.72) days for gamma distribution. We also applied the estimation based on the lognormal distribution and the Weibull distribution. The fitted distributions are shown in Figure 1 and the estimated parameters are reported in Table 2. We can see that, for most cases, the sample mean and sample SD agree quite well with the estimated values according to the gamma, lognormal, and Weibull distribution. From now on, in the main text, we only report sample values and fitted values from a gamma distribution.

**Table 2:** Estimated values of SI and GI.

**Group**		**Interval**	**Mean [95 CI%]**	**SD [95 CI%]**	**WAIC**
All	Lognormal	SI	6.21 (5.76, 6.70)	5.54 (4.84, 6.39)	2,758
		GI	4.72 (4.04, 5.52)	2.64 (1.91, 3.72)	316
	Gamma	SI	6.05 (5.68, 6.44)	4.32 (3.97, 4.72)	2,745
		GI	4.81 (4.13, 5.58)	2.52 (1.93, 3.32)	317
	Weibull	SI	6.07 (5.71, 6.45)	4.21 (3.90, 4.57)	2,752
		GI	4.83 (4.17, 5.55)	2.38 (1.91, 3.04)	316
Imported	Lognormal	SI	6.73 (6.04, 7.54)	6.42 (5.28, 7.88)	1,471
-First		GI	5.32 (4.46, 6.27)	1.94 (1.26, 3.04)	131
		SI^arrival^	10.95 (10.27, 11.68)	6.00 (5.27, 6.87)	1,681
		GI^arrival^	2.77 (2.32, 3.30)	1.75 (1.23, 2.56)	251
	Gamma	SI	6.52 (5.96, 7.13)	4.79 (4.25, 5.43)	1,455
		GI	5.47 (4.57, 6.45)	2.03 (1.35, 3.07)	133
		SI^arrival^	10.85 (10.24, 11.48)	5.23 (4.74, 5.79)	1,663
		GI^arrival^	2.84 (2.35, 3.39)	1.80 (1.35, 2.45)	261
	Weibull	SI	6.53 (5.99, 7.10)	4.54 (4.07, 5.1)	1,453
		GI	5.52 (4.62, 6.41)	1.92 (1.37, 2.72)	133
		SI^arrival^	10.86 (10.26, 11.47)	5.08 (4.70, 5.53)	1,665
		GI^arrival^	2.82 (2.30, 3.40)	1.90 (1.50, 2.51)	266
Local first- second	Lognormal	SI	6.05 (5.40, 6.81)	5.03 (4.08, 6.28)	994
		GI	4.51 (3.29, 6.20)	2.94 (1.60, 5.62)	90
	Gamma	SI	6.01 (5.44, 6.64)	4.15 (3.62, 4.79)	994
		GI	5.01 (3.58, 7.06)	3.17 (1.81, 5.67)	90
	Weibull	SI	6.04 (5.46, 6.67)	4.14 (3.66, 4.73)	1,000
		GI	4.95 (3.61, 6.77)	2.78 (1.73, 4.86)	90
Local second- third+	Lognormal	SI	4.34 (3.66, 5.18)	3.13 (2.27, 4.42)	286
		GI	3.77 (2.59, 5.28)	2.78 (1.49, 5.48)	90
	Gamma	SI	4.39 (3.74, 5.15)	2.76 (2.19, 3.54)	286
		GI	4.25 (2.82, 6.23)	3.13 (1.74, 6.0)	49
	Weibull	SI	4.40 (3.75, 5.13)	2.70 (2.22, 3.39)	289
		GI	4.26 (2.86, 6.08)	2.89 (1.77, 5.50)	91

*The widely applicable information criterion (WAIC) can be used to select a model: The one with a minimal WAIC value can be regarded as the best-fit model. Note that for most cases, while the mean of GI and SI are not the same, although still not that different either, since they are often within their 95% CIs, their standard deviations are clearly different. Of course, for intervals upon arrival, GI and SI should be different in definition since SI^arrival^ − GI^arrival^ ≈ IP > 0*.

To further understand the wide range of the previously reported SIs, we estimated the distribution of SIs on three subsets. For the imported-first subset $E_{1}^{0}$ with 261 events, the observed SIs have a mean at μ_SI_ = 6.50 days and an SD at δ_SI_ = 4.49 days. We estimated the mean at 6.52 (95% CI:5.96 − 7.13) and SD at 4.79 (95% CI:4.25 − 5.43) from the gamma distribution (Figure 1B). Our estimated SI of the imported-first subset is slightly smaller than, but close to, the reported value of 7.5 (6).

For the local first-second subset $E_{2}^{1}$ with 186 events, the observed SIs have a mean μ_SI_ = 5.97 days and an SD δ_SI_ = 4.31 days. We estimated the mean at 6.01 (95% CI:5.44 − 6.64) days and SD at 4.15 (95% CI:3.62 − 4.79) days from the gamma distribution (Figure 1C).

For the local second-third plus subset with 62 events, the observed SIs have a mean μ_SI_ = 4.49 days and an SD δ_SI_ = 2.79 days. We estimated the mean at 4.39 (95% CI:3.74 − 5.15) days and SD at 2.76 (95% CI:2.19 − 3.54) days for the gamma distribution (Figure 1D). The estimated SI is close to the lower bound 3.95 (3).

It is found that the estimated SI gradually decreases from 6.52 to 4.39 as generation increases. This discovery also explains to a certain degree why previous reported SIs in different papers are sometimes quite different. This result also reminds us to look into the reasons for such a trend in SI. Qian et al. (4) and Wei et al. (5) pointed out that with more and more infective cases, it is more probable that an earlier infection will happen if there are pre-symptomatic transmissions. The earlier infections will likely make SI smaller. Thus, the gradually decreasing SI leads us to examine whether or not there are pre-symptomatic transmissions.

### 3.2. Pre-symptomatic Transmissions

To check if there are pre-symptomatic transmissions, we compared the earliest exposure time of an infectee with the onset time of an infector. It was found that 135 of the 509 (26.5%) reports indicate that infectees may be infected before symptoms of infectors appear. Moreover, pre-symptomatic transmissions have occurred 57 of the 261 (21.8%) events in the imported-first subset, 46 of 186 (24.7%) events in the local first-second subset, 32 of 62 (51.6%) events in the local second-third plus subset. The ratio of pre-symptomatic transmission increases as generation increases.

### 3.3. Generation Interval

GI distribution is needed for the inference of the reproduction number (25). Often people use SI as a proxy of GI as the time of infection is not often reported in case files. In principle, SI and GI should have equal expected values since the IP time for the infector and infectee should cancel out. Consequently, GI is less studied than SI. However, GI and SI still might have different standard deviations even if they have the same mean. As we will see later, it turns out that for COVID-19, even the mean of GI and SI are slightly different, and their standard deviations are clearly different. Second. and more importantly, for epidemics with pre-symptomatic transmissions, one needs GI instead of SI since, even before the onset of symptoms, transmissions can occur already. It has recently been shown that estimates of the reproduction number are biased when ignoring the difference between SI and GI (26). Surprisingly, very few papers have studied GI of COVID-19 (3). In this work, we would like to add one more study to GI of COVID-19.

To obtain a GI value, we need exposure times of both the infector and the infectee in a transmission chain. However, exposure time is not available for many cases. Therefore, to estimate GIs, we only consider imported cases with travel history (i.e., Hubei travelers) and use the middle of their trips as their dates of exposure since people can often remember the dates of their trips much better. After that, we only obtained 67 events for estimating GI from 509 transmission chains.

**Figure 2:**
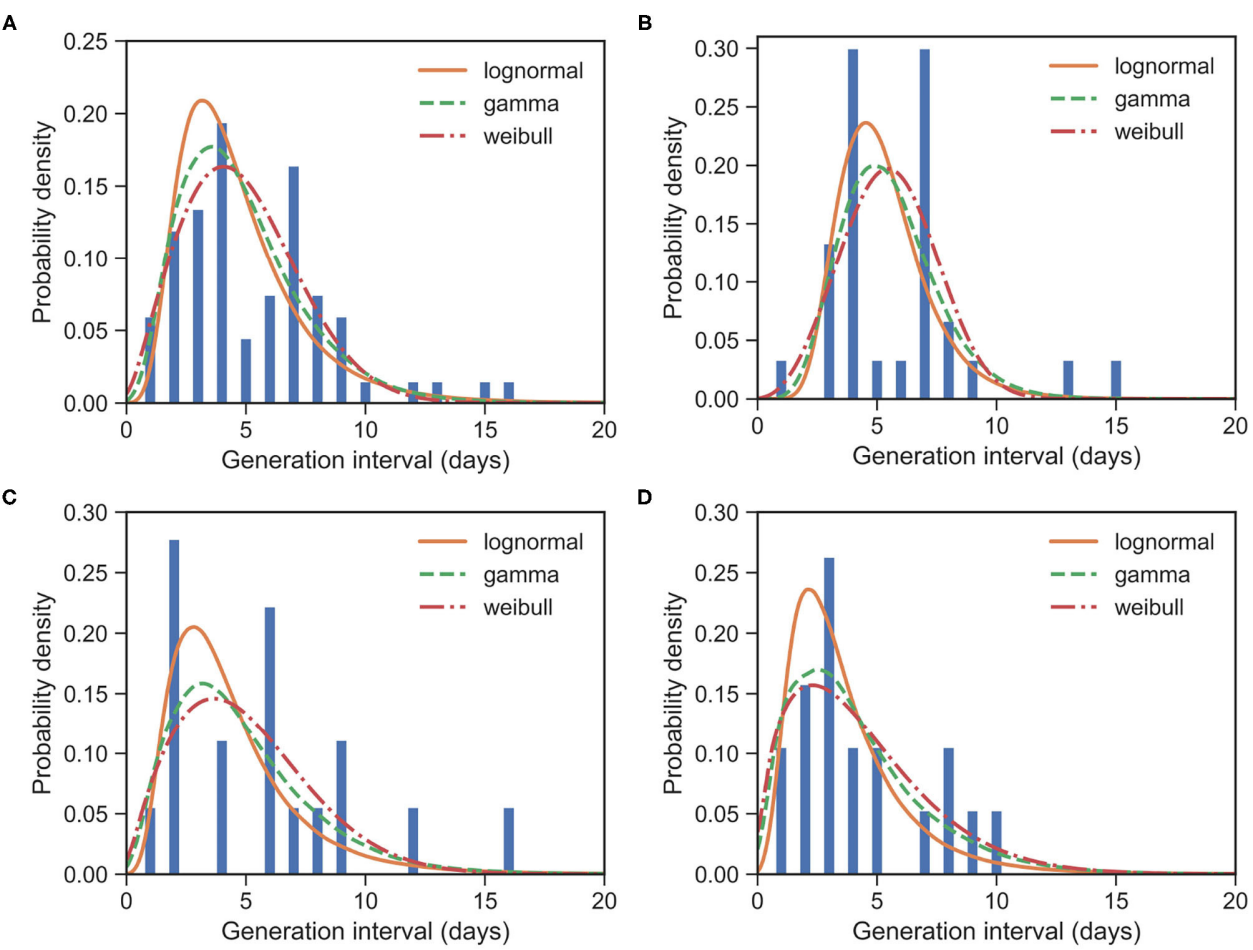
Fitted GI distributions for COVID-19 based on 67 reported transmission pairs. Bars indicate the empirical distribution of GI samples and lines indicate the fitted lognormal, gamma and Weibull distributions, respectively. **(A)** The “All” dataset (*N* = 67). **(B)** The imported-first subset $G_{0}^{1}$ (*N* = 30). **(C)** The local first-second subset $G_{1}^{2}$ (*N* = 18). **(D)** The local second-third plus subset $G_{2+}^{3+}$ (*N* = 19). Values of fitted parameters can be found in Table 2.

For the whole dataset with 67 events, the observed GIs have a mean of μ_GI_ = 5.42 days and an SD of δ_GI_ = 3.23 days. We estimated the mean at 4.81 (95% CI:4.13 − 5.58) days and SD at 2.52 (95% CI:1.93 − 3.32) days for the gamma distribution. The fitted distributions are shown in Figure 2 and the estimated parameters are reported in Table 2.

For the imported-first subset with 30 events, the observed GIs have a mean of μ_GI_ = 5.87 days and an SD of δ_GI_ = 2.96 days. We estimated the mean at 5.47 (95% CI:4.57 − 6.45) days and SD at 2.03 (95% CI:1.35 − 3.07) days for the gamma distribution.

For the local first-second subset with 18 events, the observed GIs have a mean μ_GI_ = 5.78 days and an SD of δ_GI_ = 3.99 days. We estimated the mean at 5.01 (95% CI:3.58 − 7.06) days and SD at 3.17 (95% CI:1.81 − 5.67) days for the gamma distribution.

For the local second-third plus subset with 19 events, the observed GIs have a mean μ_GI_ = 4.37 days and an SD of δ_GI_ = 2.75 days. We estimated the mean at 4.25 (95% CI:2.82 − 6.23) days and SD at 3.13 (95% CI:1.74 − 6.0) days for the gamma distribution.

The estimated mean values of GI and SI seem to be slightly different, although their confidence intervals overlap marginally. Their standard deviations are clearly different. Moreover, as the generation increases, the means of GIs decrease from 5.47 to 4.25. This is consistent with the decreasing SI, as reported in section 3.1. Of course, such a difference between GI and SI may be caused by the small sample size in our GI data, or they might indeed be different. This difference calls for further analysis, which in turn calls for more information to be provided in the reported case files. We would like to point out that such differences between GI and SI at least make it unsuitable for replacing the distribution of GI with the distribution of SI in estimating reproduction numbers, as noted already by (26).

### 3.4. Incubation Period

Depending on different sample datasets, the estimated IP in previous studies have an even wider range of 3.9 − 10.91 days (6, 8, 11–15, 17, 19, 22). Please see Table 1 for their estimated values and sample sizes. Such a large discrepancy makes it difficult to plan for public health interventions.

**Figure 3:**
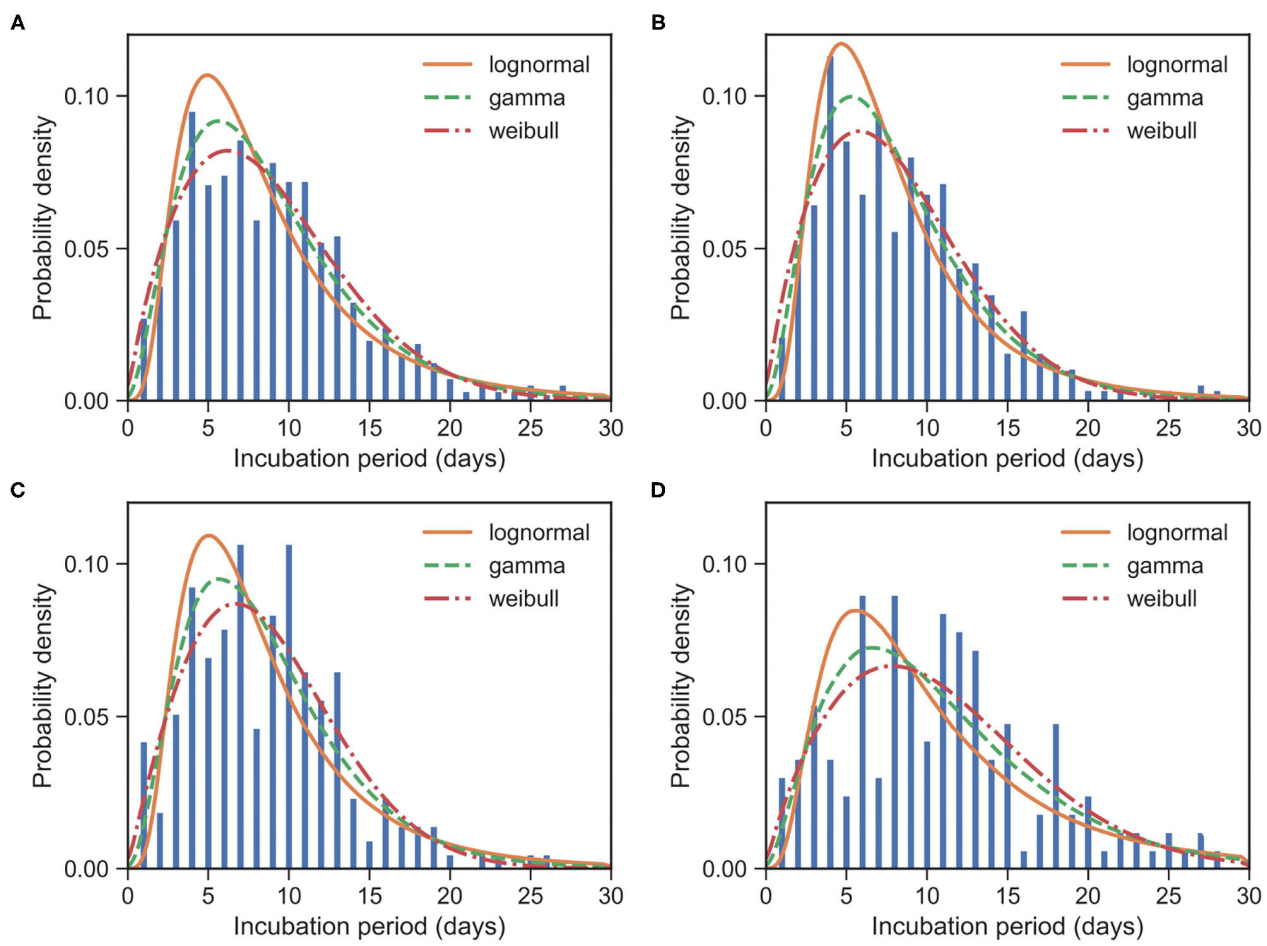
Fitted IP for COVID-19 based on 957 cases. Bars indicate the empirical distributions of IP samples and lines indicate the fitted lognormal, gamma, and Weibull distributions, respectively. **(A)** The “All” dataset (*N* = 957). **(B)** The imported cases subset (*N* = 574). **(C)** The local first-generation cases subset (*N* = 216). **(D)** The local second-plus cases subset (*N* = 167). Values of fitted parameters can be found in Table 3.

To estimate IP, we need the date of exposure and the date of symptom onset for each case. We identified 957 cases satisfying this condition from our data. From all of 957 samples, we observed μ_IP_ = 8.96 days and δ_IP_ = 5.18 days. We estimated the mean at 8.67 (95% CI:8.34 − 9.02) days and SD at 5.16 (95% CI:4.85 − 5.49) days for the gamma distribution. The fitted distributions are plotted in Figure 3 and the estimated parameters are reported in Table 3.

**Table 3:** Estimated IP values for various distributions and for various generations.

**Group**		**Mean [95 CI%]**	**SD [95 CI%]**	**WAIC**
All	Lognormal	8.80 (8.41, 9.21)	6.10 (5.58, 6.68)	5,716
	Gamma	8.67 (8.34, 9.02)	5.16 (4.85, 5.49)	5,686
	Weibull	8.69 (8.36, 9.03)	5.02 (4.77, 5.30)	5,697
Imported	Lognormal	8.11 (7.67, 8.58)	5.35 (4.78, 6.01)	3,332
	Gamma	8.06 (7.65, 8.48)	4.70 (3.33, 5.10)	3,333
	Weibull	8.08 (7.67, 8.49)	4.65 (4.34, 4.99)	3,347
Local	Lognormal	8.74 (8.0, 9.57)	5.87 (4.94, 7.03)	1,277
first	Gamma	8.59 (7.95, 9.29)	4.86 (4.31, 5.51)	1,258
generation	Weibull	8.59 (7.97, 9.23)	4.60 (4.16, 5.13)	1,255
Local	Lognormal	11.01 (9.84, 12.36)	8.34 (6.77, 10.41)	1,086
second-plus	Gamma	10.79 (9.81, 11.86)	6.65 (5.78, 7.70)	1,068
generation	Weibull	10.80 (9.88, 11.78)	6.15 (5.47, 7.0)	1,063
IP^arrival^	Lognormal	6.90 (6.60, 7.23)	6.25 (5.76, 6.80)	8,124
	Gamma	6.67 (6.43, 6.92)	4.76 (4.52, 5.01)	8,062
	Weibull	6.68 (6.45, 6.93)	4.62 (4.41, 4.85)	8,074

Again, we divided the dataset into three subsets, the imported cases with travel history (*X*^0, *T*^) (i.e., Hubei travelers), the local first-generation cases (*X*^1^), and the local second-plus generation cases (*X*^2+^). For the imported subset with 574 cases, the observed IPs have a mean of μ_IP_ = 8.51 days and an SD of δ_IP_ = 4.94 days. We estimated the mean at 8.06 (95% CI:7.65 − 8.48) days and SD at 4.70 (95% CI:4.33 − 5.10) days for the gamma distribution. We take the exposure date of the imported cases with travel history to be the middle of their trips since one can often remember dates of traveling accurately. Moreover, for most imported cases, their traveling times are often quite short.

For the local first-generation (*X*^1^) subset with 216 cases, the observed IPs have a mean μ_IP_ = 8.69 days and an SD of δ_IP_ = 4.71 days. We estimated the mean at 8.59 (95% CI:7.95 − 9.29) days and SD at 4.86 (95% CI:4.31 − 5.51) days for the gamma distribution.

For the local second-plus generation (*X*^2+^) subset with 167 cases, the observed IPs have a mean of μ_IP_ = 10.86 days and an SD of δ_IP_ = 6.08 days. We estimated the mean at 10.79 (95% CI:9.81 − 11.86) days and SD at 6.65 (95% CI:5.78 − 7.70) days for the gamma distribution.

### 3.5. Intervals Upon Arrival for Imported Cases

Sometimes, for imported cases in particular, knowing after their arrival how long they will typically show symptoms, infect locals, and also when the local infectees, who are infected by the imported cases, will show symptoms, can also be informative for decision-makers of intervention strategies. Therefore, in this work, we also show our results on these statistics.

**Figure 4:**
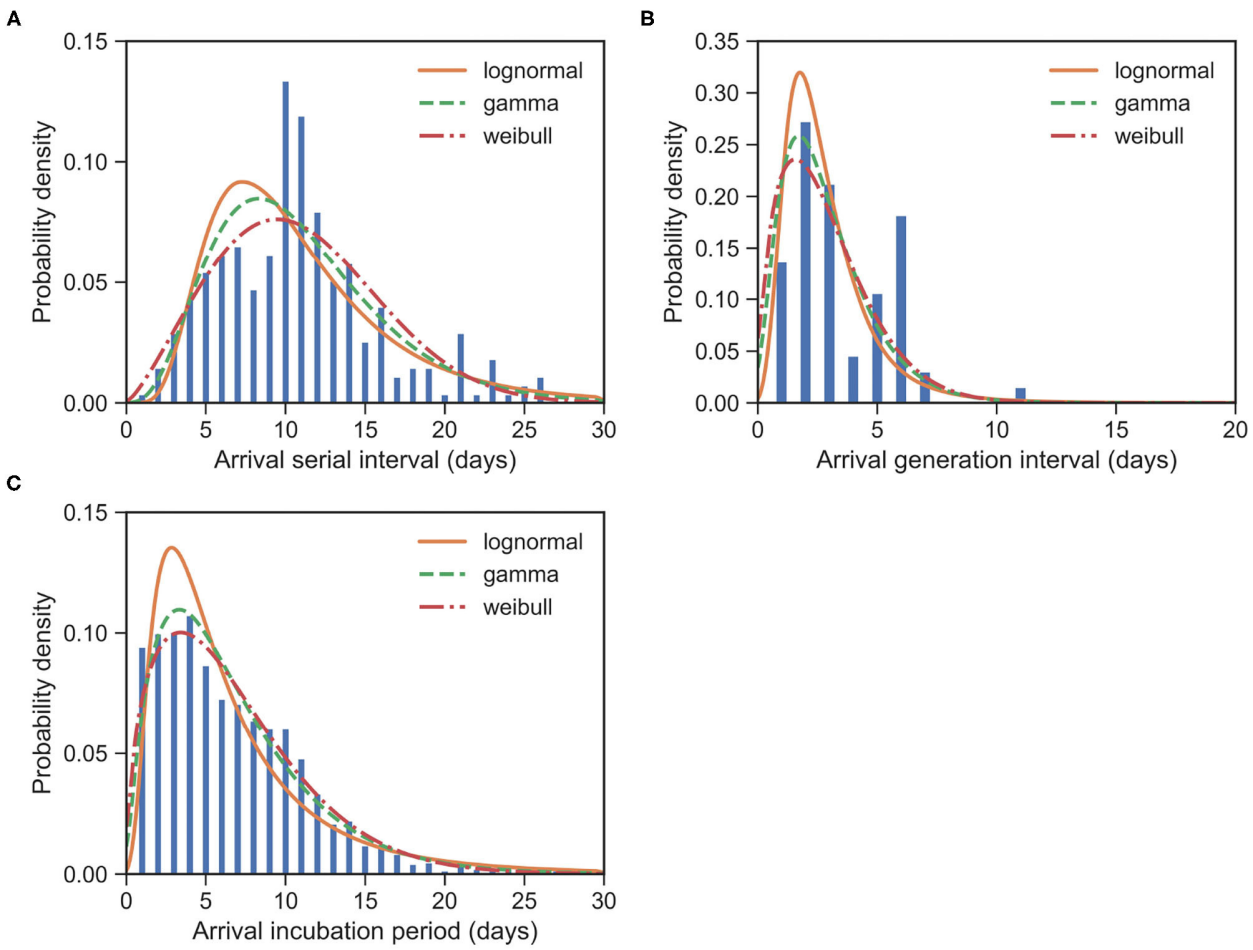
Fitted distributions of the various intervals upon arrival for imported cases of COVID-19. Bars indicate the empirical distributions and lines indicate the fitted lognormal, gamma, and Weibull distributions, respectively. **(A)** SI upon arrival (SI^arrival^) (*N* = 277). **(B)** GI upon arrival (GI^arrival^) (*N* = 66). **(C)** IP upon arrival (IP^arrival^) (*N* = 1, 443). Values of fitted parameters are reported in both Tables 2, 3.

The serial interval upon arrival (SI^arrival^) is defined as the interval between the date that an imported case arrives at the reporting city and the date that the infectee, infected by the imported case, shows symptoms. For 277 transmission events, the observed SI^arrival^s have a mean of $\mu_{{\text{SI}}^{\text{arrival}}}=10.83$ days and an SD of $\delta_{{\text{SI}}^{\text{arrival}}}=5.08$ days. We estimated the mean at 10.85 (95% CI:10.24 − 11.48) days and SD at 5.23 (95% CI:4.74 − 5.79) days for the gamma distribution (Figure 4A). The estimated SI^arrival^ is reported in Table 2.

The generation interval upon arrival (GI^arrival^) is defined as the interval between the date that an imported case arrives at reporting city and the date that he/she infects others. For 66 transmission events, the observed GI^arrival^s have a mean of $\mu_{{\text{GI}}^{\text{arrival}}}=3.50$ days and an SD of $\delta_{{\text{GI}}^{\text{arrival}}}=2.05$ days. We estimated the mean at 2.84 (95% CI:2.35 − 3.39) days and SD at 1.80 (95% CI:1.35 − 2.45) days for the gamma distribution (Figure 4B). The estimated GI^arrival^ is reported in Table 2. In definition, SI^arrival^ is more or less the summation of GI^arrival^ and IP, which is SI^arrival^ − GI^arrival^ ≈ IP > 0, unlike the relation between the usual SI and GI, SI ≈ GI.

The incubation period upon arrival (IP^arrival^) is defined as the interval between the date that an imported case arrives at the reporting city and the date that the imported case shows symptoms. For 1, 443 cases, the observed IP^arrival^s have a mean of $\mu_{{\text{IP}}^{\text{arrival}}}=6.67$ days and an SD of $\delta_{{\text{IP}}^{\text{arrival}}}=4.71$ days. We estimated the mean at 6.67 (95% CI:6.43 − 6.92) days and SD at 4.76 (95% CI:4.52 − 5.01) days for the gamma distribution (Figure 4C). The estimated IP^arrival^ is reported in Table 3. It is found that IP^arrival^ is larger than GI^arrival^. This indicates again that pre-symptomatic transmissions do occur.

## 4. Conclusion and Discussion

In this paper, we first estimated SI based on 509 transmission events, which are divided into three subsets, including imported-first subset $E_{1}^{0}$, local first-second subset $E_{2}^{1}$ and local second-third plus subset $E_{3+}^{2+}$. It is found that the estimated SI decreases with the number of generations and they are ${\text{SI}}_{1}^{0}=6.52\pm 4.79$, ${\text{SI}}_{2}^{1}=6.01\pm 4.15$, ${\text{SI}}_{3+}^{2+}=4.39\pm 2.76$, respectively. We also found that pre-symptomatic transmissions likely occurred in 135 events out of 509 events (26.5%).

We then estimated GI in the three subsets. It was also found that the estimated GI decreases as the generation increases, and they are ${\text{GI}}_{1}^{0}=5.47\;\pm \;2.03$, ${\text{GI}}_{2}^{1}=5.01\;\pm \;3.17$, ${\text{GI}}_{3+}^{2+}=4.25\;\pm \;3.13$, respectively. We would like to point out that there are small differences between the means of GI and the corresponding SI, and clear differences between their standard deviations. This, together with the existence of pre-symptomatic transmissions, makes it necessary to use GI in estimating reproduction numbers rather than SI.

Next, we estimated IP of different groups of cases. It was found that the estimated IPs are IP^0, *T*^ = 8.06 ± 4.7 days for 574 Hubei travelers, IP^1^ = 8.59 ± 4.86 for 216 local first-generation cases, and IP^2+^ = 10.79 ± 6.65 days for 167 local second plus-generation cases.

Moreover, we estimated the SI, GI, and IP upon arrival of the imported cases at the reporting city. It was found that the imported cases will show symptoms after IP^arrival^ = 6.67 ± 4.76 days of arrival in reporting cities and will infect others after GI^arrival^ = 2.84 ± 1.8 days. The difference between these two intervals also indicates that pre-symptomatic transmission is likely to occur. Finally, it was found that the local first-generating cases will show symptoms after SI^arrival^ = 10.85 ± 5.23 days imported cases arrived at the reporting cities.

Providing statistics for various generations of cases, so that in further studies better models can be established, for example, by making use of different values of transmission parameters for different generations, is the main contribution of this work. Our results also explain to a certain degree why in previous studies values of those estimated parameters span across a wide range. For the imported cases, in particular, we reported SI, GI, and IP upon their arrivals. This study can be meaningful for both planning intervention and modeling epidemics. Furthermore, one should note that for epidemics with pre-symptomatic transmissions, when estimating the basic and the time-varying reproduction number, GI should be used instead of SI.

There are several limitations in this study. Our data is restricted to online reports from only 20 provinces in China. The content of epidemiological investigation reports from different provinces varies a lot. Many case reports do not have exposure date and an infector ID, which is crucial in epidemics modeling. Thus, while admitting this limitation, here we also call for designing/utilizing a standard format of the case reports, countrywide, or even worldwide. Our sample size, especially on generation interval, is still very small. Our results for GI and GI upon arrival are therefore not as reliable as the ones for SI.

## Data Availability Statement

The datasets generated for this study can be found in online repositories. The names of the repository/repositories and accession number(s) can be found at: https://github.com/Bigger-Physics/COVID19-si.

## Author Contributions

JW, KL, and MW designed this study, while ML and YS cleaned the data and performed the analysis. All authors wrote the manuscript together.

## Conflict of Interest

The authors declare that the research was conducted in the absence of any commercial or financial relationships that could be construed as a potential conflict of interest. The reviewer YW declared a shared affiliation, with no collaboration, with several of the authors, KL, YS, MW, and JW to the handling editor at the time of review.
